# Comparative Physiological, Proteomic, and Metabolomic Insights into a Promising Low-Pruning Mulberry Cultivar for Silkworm Rearing

**DOI:** 10.3390/ijms252413483

**Published:** 2024-12-16

**Authors:** Yan Liu, Zhiqiang Lv, Jia Wei, Peigang Liu, Meiliang Pan, Huanyan Ma, Tianbao Lin

**Affiliations:** 1 Institute of Sericulture and Tea, Zhejiang Academy of Agricultural Sciences, Hangzhou 310021, China; lvzq@zaas.ac.cn (Z.L.); weijia@zaas.ac.cn (J.W.); liupeigang@zaas.ac.cn (P.L.); 2 Department of Agriculture and Rural Affairs, Zhejiang Provincial Center for Agricultural Technology Extension, Hangzhou 310020, China; pmlcsk@163.com (M.P.); mahuanyan1111@163.com (H.M.)

**Keywords:** low-pruning cultivation, proteomics, metabolomics, mulberry, sericulture

## Abstract

Mulberry (*Morus* spp.) is an economically significant plant in the production of silk through feeding leaves to silkworm larvae. Traditional silkworm rearing is heavily labor-intensive, particularly in leaf collection, which leads to low efficiency and impedes the development of sericulture. Here, to assess the feasibility and effectiveness of a novel low-pruning mulberry cultivar, ZJ1, in the silkworm rearing industry, a comprehensive investigation integrating physiological, proteomic, and metabolomic analyses was conducted in comparison with the traditionally high-pruning cultivar, N14. The low-pruning mulberry variety ZJ1 exhibited a notable increase in annual leaf yield of 43.94%, along with a significant enrichment of serine and isoleucine contents, in contrast to those of the high-pruning variety N14. Through iTRAQ proteomics and LC-MS/MS metabolomics analyses, a total of 561 reduced and 803 increased differentially expressed proteins (DEPs), as well as 332 differential expressed metabolites (DEMs) in positive ions and 192 DEMs in negative ions, were identified in the ZJ1 group relative to the N14 group, respectively. The observed features in amino acid profiles and the enrichment of the sucrose-related metabolic pathway provided interesting insights for future endeavors in mulberry variety improvement and the optimization of silkworm diet formulations. Collectively, the low-pruning cultivar ZJ1, characterized by its rapid growth, high leaf productivity, and suitability for mechanized operations, is expected to be an efficient substitute in improving the future sericultural industry, especially in urbanized and industrialized regions.

## 1. Introduction

Mulberry (*Morus* spp.) is a fast-growing and economically important perennial tree that is widely cultivated in China, India, and numerous other countries around the world. The diverse components of mulberry, along with its positive environmental impacts, ensure the broad utilization of mulberry trees in pharmaceuticals, husbandry, ecology, and sericulture for the rearing of silkworms in particular [1,2,3]. The silkworm (*Bombyx mori* L.) is an essentially monophagous and host-plant-specific insect that feeds solely on mulberry leaves and has been domesticated for thousands of years to produce raw silk from its cocoon [4]. Silkworm silk is an easily available renewable protein material with excellent qualities. The toughness of silk fiber and its unusual combination of high strength and expansibility have not been surpassed by any synthetic materials to date [5]. Growing mulberry trees, rearing silkworms, and reeling silk have all along been regarded as ecofriendly that play valuable roles in global economic development [6,7].

**Figure 1:**
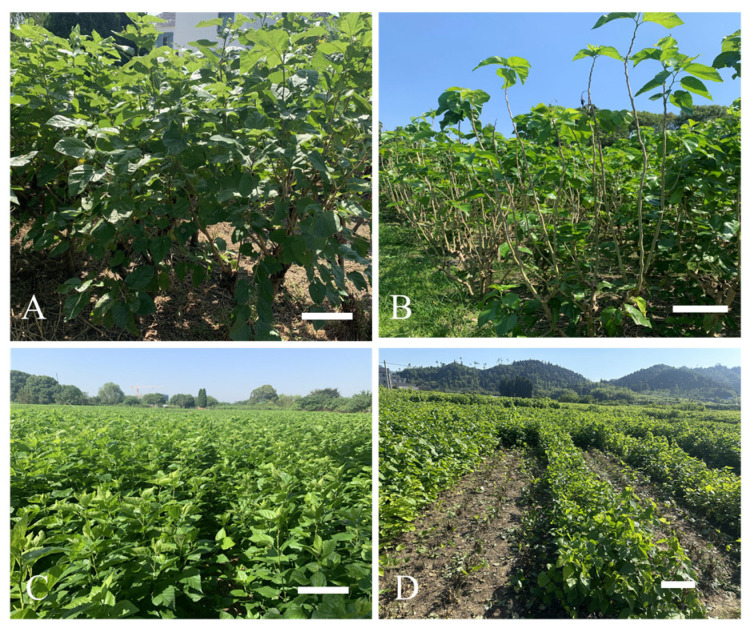
Field growth and harvest performance phenotype of different cultivated mulberries. The cultivation of high-pruning mulberry N14 before (**A**) and after (**B**) a field harvest. The cultivation of low-pruning mulberry ZJ1 before (**C**) and after (**D**) a field harvest. Scale bar = 50 cm.

In the past several decades, to facilitate leaf picking for silkworm rearing, mulberry has typically been cultivated in a shrubby way with high pruning. This traditional high-pruning cultivation model requires at least three years to establish a viable plantation and put it into production. Additionally, the sericulture industry used to be a labor-intensive rural industry as it relies extremely on manual labor at every stage of the process, including leaf picking, silkworm feeding, and cocoon collection. Currently, with the growing scarcity of labor and the limitation of arable mulberry fields, production costs in the sericultural industry are increasing, and the comparative economic benefit is declining. There is an urgent need for a new alternative model to ensure the sustainable development of the sericultural industry. Numerous factors control plant growth, such as genotype and cultivation management [8,9,10]. In the past decades, many studies have shown that hybrids derived from a cross between two inbred parental lines often display superior traits compared to those of the parents [11]. The increasing application of hybrid varieties (e.g., hybrid rice) has been widely adopted by farmers [12]. A recent hybrid mulberry variety (e.g., Zhesangza 1#, ZJ1) exhibits excellent properties, including easy planting, fast growth, easy regeneration, and vigorous growth even after low pruning, which enables it to be mechanically reaped from the base of the stem. This hybrid mulberry thereby exhibits potential advantages of large scale and labor savings in sericulture applications (Figure 1). In addition, pruning is a crucial management factor that modulates plant architecture, promotes stem growth, and thus encourages biomass production. Many researchers have implemented pruning experiments in trees and food crops [9,13], yet various foliage and branch biomass alterations have been noted among a set of tree species in response to different intensities and frequencies of pruning [14]. The available details regarding the low-pruning technique of mulberry are limited. It is essential to examine the effectiveness and feasibility of raising silkworms using this novel cultivation model case by case.

In recent years, multiple omics analyses, including proteomics and metabolomics, have been established for exploring many regulatory mechanisms in various organisms. Proteomics provides a powerful tool for describing complete proteomes at the organelle, cell, or tissue levels. Meanwhile, metabolomics focuses on endogenous metabolites synthesized through a series of multiple enzymatic steps from various biochemical pathways in the plant genome [15,16]. Both proteomics and metabolomics are increasingly popular in assessing plant phenotypes and genetic diversity and have been extensively applied to plants such as peach [17], jujube [18], loquat [19], and citrus [20]. The combination of proteomics and metabolomics has received much attention, as they can mutually verify and complement each other, vividly reflecting the changes that occur during plant growth and development, especially in non-model organisms [21,22,23]. Currently, the regulatory mechanisms and alterations within the mulberry and silkworm systems under the new sericulture model still remain largely unclear. The application of proteomics and metabolomics would facilitate comprehension of this innovative cultivation approach.

To extensively investigate the characteristics of mulberry leaves under different cultivation modes and their effects on silkworm rearing, an integrated approach combining physiological, proteomic, and metabolomic analyses was employed in the current study. Through this approach, we aim to gain insights into the novel cultivation mode that suits future mechanical operations in the sericultural industry.

## 2. Results

### 2.1. Low-Pruning Cultivation Mulberry Showed Characteristics of High Yield and Mechanical Harvestability

Mulberry was cultivated traditionally in a high-pruning shrubby way for leaf picking and silkworm rearing, as exemplified by the popular variety N14 in China. In the context of the sericultural industry, N14 is typically planted at a density of around 800 strains per acre. Its trunk was trimmed to about approximately 60 cm in height, with two to three limbs were retained, thereby facilitating the growth of the foliage. To ensure the emergence of newly born leaves suitable for silkworm rearing, all limbs were cut in summer. Subsequently, each newly grown limb in autumn was pruned back to a length of about 1.5 m in winter. The mature leaves of N14 were predominantly hand-plucked for silkworm rearing (Figure 1A,B). Conversely, to align with the trend of large-scale factory-based production and reduce the heavy reliance on manual labor, a novel hybrid variety like Zhesangza 1 (ZJ1) could be harvested through a low-pruning method from the base of the stem using a supporting machine, as shown in Figure 1. In comparison to N14, ZJ1 exhibited a remarkable 43.94% increase in annual leaf yield under the same standard management conditions (Table 1). Interestingly, even when high-pruning mulberry varieties such as N14 were cultivated in a low-pruning way, their annual available yield was merely 89.48% of that of the specialized low-pruning cultivated varieties [24], which might be attributed to the more prosperous sprout capacity of the latter. Consequently, these specialized low-pruning cultivars exhibited superior prospects for future production. Taking the Zhejiang province, which features a subtropical monsoon climate and is situated at approximately 30 degrees north latitude and 120 degrees east longitude, as an example for promoting the cultivation of low-pruning mulberry, it is recommended to plant 4000 strains per acre with a uniform planting layout suitable for mechanical harvesting. The recommended time for the first cutting time is from 10 May to 30 May in spring, and the second cutting is from 30 June to 20 July, the third cutting time is from 10 August to 30 August, and 20 September to 10 October is favored as the fourth harvesting period.

**Figure 2:**
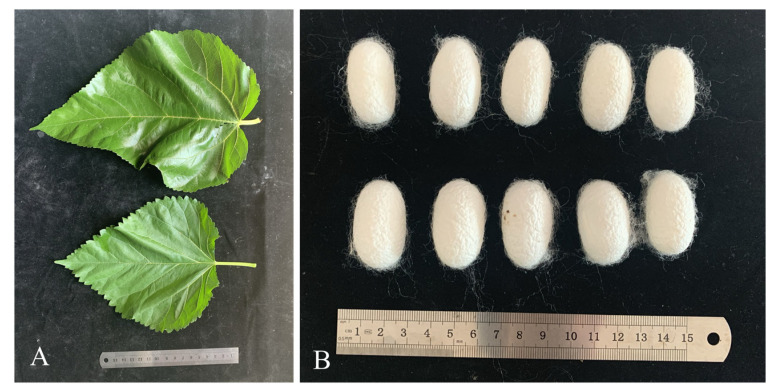
Comparison of the cocoons produced by silkworms reared on different cultivated mulberry leaves. (**A**) Phenotypic comparison of the leaves of high-pruning (upper) and low-pruning (bottom) mulberries, respectively. (**B**) Silkworm cocoons produced by feeding high-pruning mulberry leaves (upper) and low-pruning mulberry leaves (bottom), respectively.

In the present study, the average leaf size of N14 was 25 cm in length and 19 cm in width. In comparison, for ZJ1, the average leaf length was 20 cm and the average width was 16 cm. The leaves of ZJ1 exhibited a lower level of brightness and luster compared to those of N14. To better understand the physiological parameters of low-pruning mulberry, the water content, chlorophyll content, total protein, and amino acid components were analyzed, respectively. The results showed that there was a significant distinct leaf phenotype as illustrated in Figure 2. With the exception of serine and isoleucine, the protein and amino acid contents did not exhibit a statistically significant difference (Table 2). 

### 2.2. Impact of Low-Pruning Mulberry ZJ1 on the Production Performance in the Silkworm Rearing Industry

To understand the feasibility of low-pruning cultivated mulberry in the silkworm rearing industry, a total of 2400 silkworms (HK5) were randomly grouped and reared with high-pruning type N14 and low-pruning type ZJ1 leaves, respectively, throughout the entire fifth-instar larvae stage. All tested silkworm larvae grew normally without significant differences between the two groups. One week after pupation, the cocoon number, cocoon weight, and cocoon shell weight in the two groups were investigated. As shown in Table 3, silkworms fed with low-pruning ZJ1 mulberry leaves in spring had a significantly lower whole cocoon weight by 1.17%, compared to those fed with N14 leaves. However, it was interesting that the ZJ1 forage group resulted in a significantly higher cocoon shell ratio by 1.52% than that in N14-treated group. This indicated that low-pruning ZJ1 mulberry leaves might be more favorable for the formation of cocoon silk. Together with the measurement across both seasons, it was indicated that the low-pruning cultivated ZJ1 leaves benefited production performance in the silkworm rearing industry.

### 2.3. iTRAQ Comparison of Leaf Protein Profile from the Two Kinds of Cultivated Mulberry Varieties

**Figure 3:**
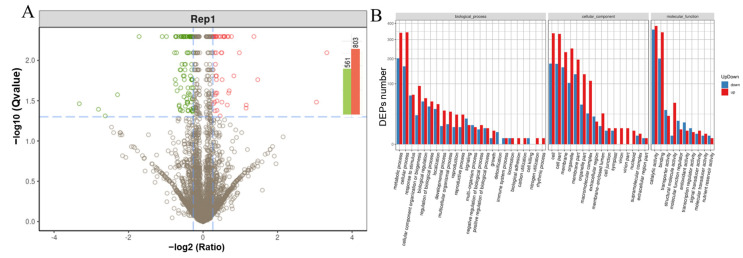
Identification and ontological classification of the differentially expressed proteins in low-pruning mulberry leaves. (**A**) Volcano plot of differentially expressed proteins, depicting the log2 fold-change (x-axis) versus the −log10 Qvalue (y-axis, representing the probability that the protein is differentially expressed). Qvalue < 0.05 and Foldchange > 1.2 are set as the significant thresholds for differential expression. The red and green dots indicate points of interest that display both large-magnitude fold-changes as well as high statistical significance. Dots in red mean significantly upregulated proteins which passed the screening threshold. Dots in green mean significantly downregulated proteins which passed the screening threshold. And gray dots are non-significantly differentially expressed proteins. (**B**) Gene ontology analysis of differentially expressed proteins. The x-axis displays the protein count; the y-axis displays the GO term.

**Figure 4:**
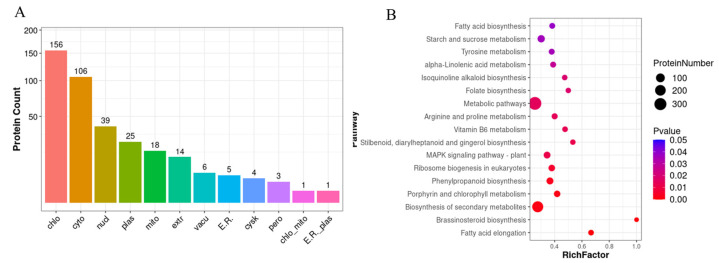
Subcellular localization and enriched functional classification of DEPs in low-pruning mulberry leaves based on KEGG pathways. (**A**) Subcellular localization prediction of DEPs in low-pruning mulberry leaves. (**B**) Statistics of the top 20 enriched pathways of DEPs in each pairwise comparison.

To explore the protein profile, iTRAQ was performed to enhance our understanding by comparing the leaves from two different cultivated strains. A total of 27,205 peptides and 5671 proteins were identified with a false discovery rate (FDR) of 1%. The molecular weight of most identified proteins was 20–40 kDa. Most peptides were 6–15 amino acids. Among them, cut-offs of 1.2-fold and 0.83-fold were established to ascertain the upregulation and downregulation of proteins. Consequently, a total of 561 downregulated and 803 upregulated proteins were identified in the ZJ1 group in comparison to the N14 group. Through gene ontology (GO) functional analysis of these differentially expressed proteins (DEPs), metabolic process, cell, and catalytic activity were assigned as the primary categories of proteins annotated in biological process, cellular component, and molecular function, respectively (Figure 3). Subcellular localization analysis demonstrated that the majority of DEPs were located in the chloroplast, cytoplasm, and nucleus (Figure 4A). Through functional analysis by KEGG pathway classification, a total of 121 different metabolic pathways were revealed. Among these, the two most enriched categories were assigned to starch and sucrose metabolism, as well as phenylpropanoid biosynthesis in addition to the global and overview maps pathway (Figure 4B). The top 20 increased and decreased expression proteins are listed in Table S1. These results suggested that starch- and sucrose-related metabolism might be involved in responding to these two different cultivation patterns and their disparities in silk performance.

### 2.4. LC-MS/MS Metabolomics Analysis of the Leaves of Two Mulberry Cultivars

**Figure 5:**
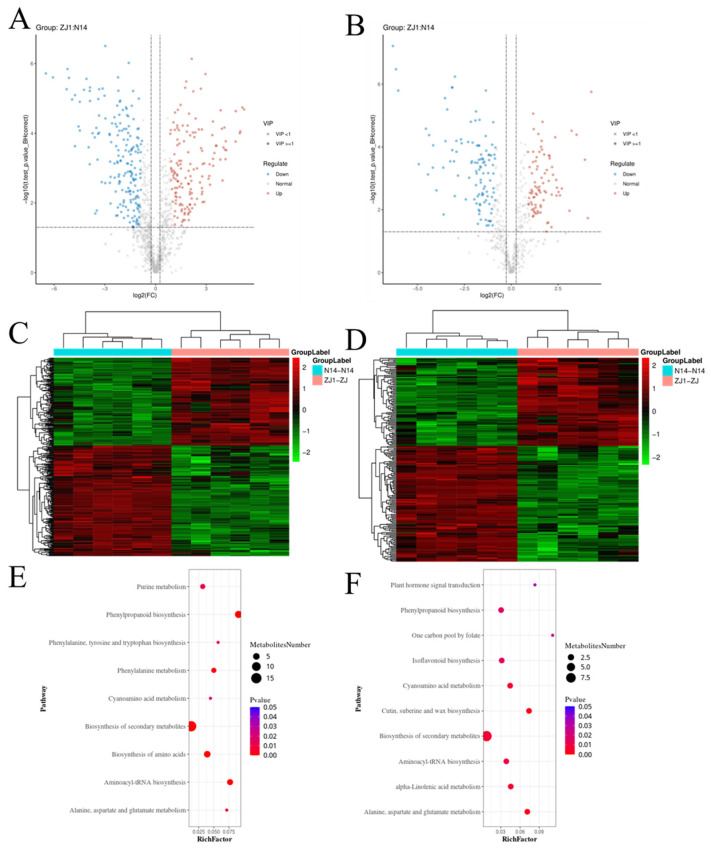
Metabolomics analysis of low-pruning mulberry leaves and high-pruning mulberry leaves. Volcano plot (**A**,**B**), heatmap (**C**,**D**), and KEGG enrichment analysis (**E**,**F**) showing differently expressed metabolites (DEMs) in low-pruning ZJ1 leaves compared to those in high-pruning N14 leaves. The left line, including panels (**A**,**C**,**E**), shows the DEMs in positive ion mode. On the right panel, including panels (**B**,**D**,**F**), the DEMs in negative ion mode are displayed.

To further explore the metabolic changes between the leaves of two cultivars, each group with six biological replicates was set up and the changes were determined by LC-MS/MS. A total of 1672 metabolites with identification information were identified, including 1118 metabolites in positive ion mode (Pos) and 554 metabolites in negative ion mode (Neg). Principal component analysis (PCA) of the dataset showed that PC1 (52.35%) and PC2 (13.92%) in Pos mode and PC1 (54.27%) and PC2 (12.36%) in Neg mode were the two main components (Figure 5A). The differentially expressed metabolites (DEMs) were then screened according to the following criteria: (1) the VIP values of the first two principal components of the PLS-DA model ≥ 1; (2) fold-change ≥ 1.2 or ≤0.83; (3) q-value < 0.05. There were 332 DEMs in Pos mode and 192 DEMs in Neg mode (Table S2) in ZJ1 samples compared to N14 samples. These DEMs were subsequently visualized through the volcano plot (Figure 5B). To analyze the differential metabolites, log2 conversion and zero-mean normalization were used as shown in Figure 5. Hierarchical clusters and the Euclidean distances were used for distance calculation. Furthermore, metabolic pathway enrichment analysis showed that metabolic pathways such as the biosynthesis of secondary metabolites, phenylpropanoid biosynthesis, and aminoacyl-tRNA biosynthesis had significant responses to the differences between the two types of mulberry leaves (Figure 5).

### 2.5. Integrated Correlation Analyses of Proteomics and Metabolomics Data

**Figure 6:**
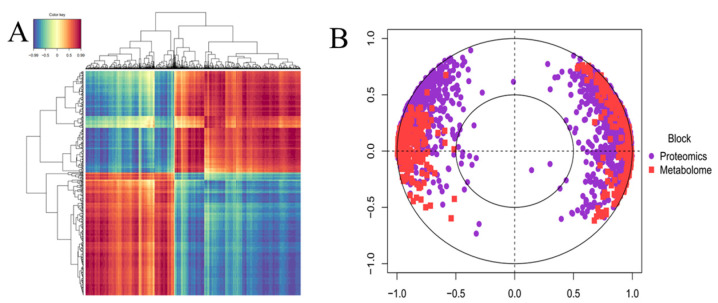
Integrated correlation analyses of proteomics and metabolomics data. (**A**) Heatmap showing correlation clustering of DEPs and DEMs identified in low-pruning mulberry leaves compared to that in high-pruning mulberry leaves. Each row represents a differential metabolite and each column represents a differential protein, with blue representing a negative correlation and red representing a positive correlation. (**B**) Splsda analysis of the correlation between differential proteins and differential metabolites in low-pruning mulberry leaves compared to that in high-pruning mulberry leaves. Each dot in the circle represents a protein, and each square represents a metabolite. An acute angle between differential proteins and differential metabolites stands for a positive correlation, while an obtuse angle stands for a negative correlation. The greater the length from the differential metabolites and differential proteins to the center of the circle, the stronger the relationship and vice versa.

A pronounced correlation was observed in the differentially expressed proteins (DEPs) and differentially expressed metabolites (DEMs) after being analyzed using the bloc.splsda function in the mixOmics package as described previously (Figure 6A) [25]. The reliability of the partial least squares–discriminant analysis (PLS-DA) model was assessed using a permutation test. The results were visualized by plotVar as shown in Figure 6B. The most closely associated metabolites and proteins included lipid-transfer protein, cell wall, and auxilin-related protein. Metabolites with strong correlations with proteins included deguelin, camphanic, and syringaresinol, representing characteristics such as different species and cultivation models.

## 3. Discussion

### 3.1. Low-Pruning Hybrid Variety ZJ1 Exhibited Superior Performance in Mulberry Cultivation and Silkworm Rearing

Mulberry planting and silkworm rearing is a traditional industry originating from China. It plays important roles in the agricultural systems of China, and in some other countries with the spread of the Silk Road [6]. In the past few decades, sericulture production has mainly relied on manual operations, especially in the processes of leaf picking and leaf feeding to silkworms, which is usually accompanied by high labor intensity and low production efficiency. Finding a mulberry variety suitable for mechanical harvesting is one of the effective strategies to meet the needs of modern sericulture. In the present study, compared with the traditional high-pruning mulberry cultivation model, the hybrid mulberry variety ZJ1 cultivated with the low-pruning method demonstrated several unique advantages. First, the low-pruning mulberry cultivation model produced an increase in annual output by 43.94% compared with the traditional high-pruning grafted mulberry variety, verifying the characteristics of fast growth and high yield of low-pruning mulberry as reported by Feng et al. (2022) [26]. Second, the low-pruning mulberry cultivation model exhibited a high harvesting efficiency and remarkable labor-saving characteristics. Owing to its suitability for mechanical harvesting, the leaf-picking efficiency of the low-pruning mulberry cultivation mode was 15.32 times greater than that of the traditional high-pruning cultivation mode, as documented in [26,27]. Meanwhile, during the silkworm rearing process, the foliage of the low-pruning ZJ1 mulberry withered more slowly compared to that of the high-pruning N14, which facilitated extending the feeding interval. Concurrently, an interesting finding revealed that there was a lesser growth of weeds in the low-pruning mulberry field. It was deduced that this was primarily attributable to the dense planting and vigorous growth of the low-pruning mulberry. The dense foliage closely blanketed the soil, leaving minimal space for weeds to thrive. As reported by Qian et al. (2021) [27], less labor was required for weed removal to guarantee the mulberry yield.

### 3.2. Diet Amino Acids Composition Affected the Performance and Yield of Silkworm Silk

The silkworm is a high-value insect that produces silk through secretion by a silk gland. Silkworm silk is an ultra-long natural protein fiber that can be used in the manufacture of luxurious textiles, medicine, bioengineering, sensors, optics, and electronics [28,29]. Different nutritional components of the mulberry leaf diet, mainly proteins and other nutrients in combination with a number of micronutrients, have an impact on silk performance and yield [30]. Among them, mulberry leaf proteins are responsible for over 70% of silk production and affect cocoon production [5]. Silk consists of a filament core protein, termed fibroin, and a glue-like coating substance formed of sericin proteins. This protein is extracted from the silkworm cocoons (particularly *Bombyx mori*) and is mainly composed of amino acids like glycine, serine, aspartic acid, and threonine [31]. Feed supplementation with mulberry leaves fortified by amino acids (alanine, glycine, and serine) was previously noted to have a positive impact on the commercial and biological characteristics of silkworms [32]. Serine is involved in the regulation of cross-links between the fat body and the silk gland. It can also regulate nutrient absorption and inhibitory neurotransmitter signaling [33]. Serine has the potential to significantly increase the commercial traits of silkworms including cocoon weight, cocoon length, cocoon width, percentage shell ratio, and percentage fibroin content as compared to controls as previously reported. Our results were in accordance with Murugesh et al. (2022) and Muzamil et al. (2023), who also reported improvements in biological traits and cocoon parameters in larvae fed with serine [32,34]. In addition, isoleucine was previously reported to be abundant in silkworm eggs [35]. Its degradation pathways might play a significant role in the heat stress response in mulberry (*M. alba*) [36]. Nelson et al. (2024) reported the critical role of isoleucine in modulating Yap signaling in cardiomyocytes under nutrient-deprivation conditions [37]. Our study found a relatively high content of isoleucine in the low-pruning hybrid mulberry ZJ1. However, its impacts on the growth and development of silkworms as well as on the formation of cocoon silk quality remain elusive and thus necessitate further investigation and identification.

Different amino acid concentrations measured in the ZJ1 variant might play some analogous roles in silkworm development and silk performance. Based on the combination with proteomic analysis, it was further found that the 40 S ribosomal protein S14 (rpS14, L484_019160.p01), which was previously located in the 40 S subunit near the 3’ end of 18 S rRNA [38], and a heat-shock protein Hsp26/Hsp42 (L484_020233.p01) that functions in the recovery of misfolded proteins and prevents aggregation [39], were prominently increased in ZJ1. These proteins might be involved in regulating the assembly of ribosomal subunits and the production of silk proteins. However, more details such as the optimally balanced composition of amino acids, their main effects on silk yield and performance, and their regulatory mechanisms remain to be determined in the future.

### 3.3. Carbohydrate Metabolism Pathway Functioned Under Different Cultivation Models

Different growth systems may impact variables related to the transport of photoassimilates and water to varying degrees [40]. Traditionally, mulberry trees are usually pruned to facilitate leaf picking for silkworm rearing in the sericultural industry. It has been reported that pruning is beneficial to promote the sprouting and growth of tillers, which is accompanied by spatiotemporal changes in sucrose content [41]. In this study, proteomics and metabolite analysis showed that KEGG pathways of starch and sucrose metabolism, carbon metabolism, and amino sugar and nucleotide sugar metabolism were significantly enriched, indicating that sugar-related signals play a role in the response of different cultivation modes. In plants, sucrose is produced in source cells through photosynthesis and serves as a supply of fixed carbon, and it could be systemically supplied throughout the plant [42]. Previous studies have suggested two sucrose synthesis pathways. First, sucrose synthesis (SUS) reversibly catalyzes the utilization of fructose and UDP-glucose (UDPG) in the regulation of sucrose metabolism in plants [43]. SUS, as the key enzyme of sugar metabolism, affects important agronomic traits and stress responses. In the present study, two sucrose synthases (L484_011124.p01, L484_011123.p01) in low-pruning mulberry leaves exhibited levels 0.83 and 0.79 times those of high-pruning mulberry leaves, respectively. This indicated that mulberry of two different cultivation types might have different carbon allocation and wood xylem formation. Glucose-1-phosphate adenylyltransferase (L484_018572.p01) in the sucrose metabolism pathway also exhibited low expression in ZJ1 mulberry, which was 0.76 times that of high-pruning mulberry. These results might indicate that low-pruning mulberry had a lower starch accumulation as reported by Zhong et al. (2024) [44]. In the second pathway, sucrose phosphate synthase (SPS) catalyzes the synthesis of sucrose-6-phosphate from fructose-6-phosphate and UDP-glucose, and then sucrose-6-phosphatase catalyzes the synthesis of sucrose from sucrose-6-phosphate in the synthesis direction [45]. A sucrose-phosphate synthase (L484_005397.p01) protein related to this pathway was observed in ZJ1, and its expression was 0.76 times lower than that in N14. In addition, an alkaline/neutral invertase (A/N-INV, L484_008994.p01), which irreversibly decomposed sucrose into fructose and glucose and played a role in starch synthesis, abiotic stress, and other plant-life activities, was also detected to be reduced by 0.55 times in low-pruning mulberry compared to high-pruning mulberry. It has been reported that the overexpression of A/N-INV in Arabidopsis exhibited higher INV activity, increased glucose, fructose, and starch content in the leaves, and promoted plant growth [46]. In this study, a carbonic anhydrase (CA) (L484_011381.p01) involved in catalyzing the interconversion of CO_2_ and HCO^3–^ had an expression level that was 1.35 times higher in low-pruning mulberry than that in high-pruning mulberry. Weerasooriya et al. (2024) reported that carbonic anhydrases were required for the initial CO_2_ delivery into plant cells to avoid adverse growth phenotypes under low-CO_2_ conditions [47]. Another beta-fructofuranosidase (invertase) (L484_019026.p01, L484_019157.p01) involved in carbohydrate transport and metabolism exhibited an opposite trend to that mentioned above. Compared to that in high-pruning mulberry, these invertases were increased by 1.31 and 1.46 times, respectively, in low-pruning mulberry. It has been reported that carbohydrates are very important for maintaining the healthy growth of young silkworm larvae [30]. Differences in varieties and cultivation management approaches, including pruning, are likely to have an impact on the plant architecture, plant biomass, and distribution of nutritional constituents [9,48]. More in-depth research on carbohydrate metabolism in the future will help to deepen the selection, improvement, and utilization of high-quality plant varieties.

## 4. Materials and Methods

### 4.1. Experimental Materials

Two mulberry varieties, five-year-old Nongsang 14 (N14) and three-year-old Zhesangza 1 (ZJ1), were utilized as materials. N14 is popularly cultivated in the traditional high-pruning mode, whereas ZJ1 is well-known for its low-pruning cultivation. Both mulberry materials grew on the same Huzhou mulberry plantation in Zhejiang province, China, at east longitude 120°, north latitude 30°. When planting N14, the interval between each strain was 150 × 50 cm, and 800 trees were planted per acre. In contrast, the planting density of ZJ1 was about 4000 plants per acre, and the planting gap was 60 × 25 cm. All planted mulberries were consistently irrigated under a uniform water-fertilizer regimen and adhered to a standardized management protocol throughout their entire growth cycle.

To compare the leaf production of the two cultivars, 60 strains of N14 and 300 strains of ZJ1 were randomly selected, harvested, and weighed each time. Four harvests were performed annually, which were arranged to align with the four traditional seasons of silkworm rearing in a year. All mature leaves of the high-pruning mulberry N14 were harvested manually and weighed in spring (10 May in the present study). After cutting off limbs of high-pruning shrubby mulberries in mid-May, which was known as “summer pruning”, the leaves of N14 on the lower section, middle section, and the remaining upper section of the germinated limbs after summer pruning were collected and weighed on 1 July, 30 August, and 10 October, respectively. Simultaneously, all foliage of ZJ1 was pruned with a mechanical harvester, and the leaves were weighed at the same four time points as those of N14. These leaf samples were either used for silkworm rearing or stored at −80 °C for measurement assays.

### 4.2. Physicochemical Index Measurement

When measuring the water content of mulberry leaves, the leaves of two varieties (10 g of mulberry leaves per share) were accurately weighed to determine the fresh weight (FW) of the leaves. Then, the mulberry leaves were placed in an oven at 80 °C until dry. After determining the dry weight (DW), the water content was calculated using the formula: Water Content (%) = [(FW−DW)/FW] × 100, as previously reported by Weng et al. (2022) [49].

In the determination of the chlorophyll content in mulberry leaves, 0.1 g of sample leaves in each group was sliced into thin strips and placed into 10 milliliters of 95% (*V*/*V*) ethanol. After being soaked for 72 h in the dark, the absorbance of the filtered solution at 663 nanometers and 645 nanometers were measured by a Tecan Infinite 200 microplate reader. The concentration of chlorophyll a and chlorophyll b was determined as reported previously [50].

After the leaves from the two cultivars were oven-dried at 65 °C to a constant weight, respectively, the samples were ground into powder with a particle size of 100 mesh. Then, the protein content, dietary fiber, and ash content were assessed according to the national standards (https://www.antpedia.com/standard) GB 5009.5-2016 (accessed on 23 June 2017), GB 5009.88-2014 (accessed on 21 March 2016), and GB 5009.4-2016 (accessed on 1 March 2017). All analyses were performed in triplicate.

Leaf samples from different cultivars were hydrolyzed in 6 mol/L HCl for 24 h. After hydrolysis, the hydrolyzed amino acid residues were derivatized using phenylisothiocyanate as the derivatization reagent. The mixture was incubated at room temperature (25 °C) for 30 min in the dark with occasional gentle shaking to promote the reaction. Then, the amino acid composition was separated by an amino acid analyzer (S-433D; SYKAM, Eresing, Germany) according to the manufacturer’s instructions [51].

### 4.3. Silkworm Rearing

In this study, experimental larvae of the Bombyx mori strain Hua Kang No.5 (practical variety HK5) were used. All of the silkworms selected for the experiments were bred at Zhejiang Academy of Agricultural Sciences. The larvae were reared under the same environmental conditions with a temperature of 25 °C and 80% relative humidity but on two different fresh diets: mulberry leaves from the high-pruning N14 (Group N14) or the low-pruning ZJ1 (Group ZJ1) from the first day of the 5th instar. Then, the mature larvae were collected and transferred to mountages for cocooning as in previous reports [52]. After a week of pupation, the cocoon number was recorded. Subsequently, we carefully opened the whole cocoons with a sharp blade. Females and males were then identified based on their different taxonomic characters at the posterior end of the abdomen. Thereafter, 25 females and 25 males were randomly selected as replicates from each group to weigh the cocoon, pupae, and cocoon shell. Three replicates were set up in each group. The cocoon shell ratio and cocoon yield/10,000 per larvae were calculated using the following formula as previously described [52,53]:

Cocoon shell rate = cocoon shell weight/cocoon weight × 100%.

Cocoon yield per 10,000 larvae = cocoon weight ∗ cocoon shell ratio ∗ 10,000/larvae number.

### 4.4. Protein Sample Preparation and iTRAQ Labeling

Leaf samples were ground into powder in liquid nitrogen, extracted as reported in our previous study [54], and quantified using the Bradford assay with bovine serum albumin as the standard. Then, samples were digested into peptides using Trypsin Gold (Promega, Madison, WI, USA). Subsequently, the iTRAQ reagent (Applied Biosystems, Framingham, MA, USA), with 113, 114, and 115 and 116, 117, and 118 iTRAQ reagents, was used to label the peptide solutions of ZJ1 and N14 samples, respectively, according to the manufacturer’s instruction. Then, the treated peptides were mixed together and dried in a SpeedVac.

### 4.5. Peptide Separation and LC-MS/MS Analysis

After labeling, the peptides were purified by strong cation exchange (SCX) chromatography as described previously [55]. A total of 12 eluted fractions were collected, desalted with strata X, and dried in a SpeedVac. Then, the collected fractions were reconstituted to a final concentration of 0.5 μg/μL and then centrifuged at 20,000× *g* for 10 min to remove insoluble material. An aliquot of 5 μL from each sample was taken and loaded onto a nano-liquid chromatography system. Data acquisition was performed on a standard parameter setting of the TripleTOF 5600 system (AB SCIEX; Concord, ON, CA). The ion spray voltage was 2.5 kV, with nitrogen gas pressures set at 30 psi for the nebulizer and 15 psi for ≤each cycle, with a total cycle time of 3.3 s. The detector recorded signals at 4 GHz, and data from four scans were combined.

### 4.6. Protein Data Processing and Bioinformatics Analysis

Raw MS/MS data were acquired and converted into MGF files, and then protein identification was performed with the Morus alba genome database (https://morus.biodb.org/morusdb/, accessed on 12 November 2021) as previously reported by Liu et al. (2019) [54]. To reduce the probability of false peptide identification, only peptides with scores significant at the 95% confidence level were considered reliable. The identified proteins were required to match at least one unique peptide. Proteins with an abundance ratio of 1.2-fold threshold change and a *p*-value ≤ 0.05 were counted as differentially expressed proteins (DEPs). The DEPs were grouped through gene ontology (http://www.geneontology.org, accessed on 12 November 2021), and the main biological pathways involved in DEPs were classified through the KEGG database (http://www.genome.jp/kegg/, accessed on 12 November 2021) to classify the main biological pathways that the DEPs were involved in.

### 4.7. Untargeted Metabolomics Analysis

As previously described [21], metabolites were extracted from mulberry leaf tissue using a methanol/water (1:1, *V*/*V*) extraction system. The supernatants were removed and then lyophilized. The redissolved mixture was filtered and transferred into autosampler vials for LC-MS/MS analysis. Reverse-phase (RP) chromatography with both positive (RP+) and negative (RP–) electrospray ionization mode was applied to cover a wide range of metabolites. The separation and quantitative measurement of metabolites were carried out using a tandem QTRAP6500 Plus high-sensitivity mass spectrometer (SCIEX, Framingham, MA, USA) and UPLC instrument (Waters Corporation, Milford, MA, USA). The raw MS data were imported into Progenesis Q1 (Waters Corporation, Milford, MA, USA) software for automatic data processing, including retention time correction, peak selection, normalization, deconvolution, and compound identification. Only the variables with more than 50% non-zero measurement values in at least one group were retained. Compound identification of metabolites was performed by comparing MS/MS spectra with an accurate *m*/*z* value derived to match the metabolite from the BGI library < in-house MS/MS library standards [56]. Data with variable importance in projection >1 and a *p*-value ≤ 0.05 were selected to determine significantly different metabolites between different comparison groups.

MetaboAnalyst (http://www.metaboanalyst.ca, accessed on 18 February 2022) was used to enrich important metabolite pathways. The O2PLS model (https://www.omicshare.com/tools, accessed on 18 February 2022) was employed to determine related metabolites and proteins by integrating proteomic and metabolome data. Finally, core proteins were enriched through GO and KEGG pathway enrichment analysis. With FDR ≤ 0.05 as the threshold, significantly enriched GO terms and pathways were obtained [57].

### 4.8. Proteome and Metabolic Sequencing Cooperation Analysis

The integration of proteome and metabolic data was carried out using the sparse partial least squares–discriminant analysis model (sPLS-DA). Sparse PLS-DA is a multivariate monitoring method for the classification of high-dimensional bio-omics data, capable of variable selection and dimension reduction [58]. The network was exported and visualized in plotVar.

### 4.9. Statistical Analysis

Physiological data were presented as the mean ± standard error. Differences among treatments were analyzed with SPSS software (Version 22.0) using Duncan’s multiple range test at *p* ≤ 0.05.

## 5. Conclusions

This study comprehensively assessed the physiological, proteomic, and metabolic profiles across various mulberry cultivation methods, highlighting the low-pruning hybrid mulberry variety ZJ1 as a promising candidate for the sericulture industry, attributed to its rapid growth, enhanced leaf yield, adaptability to mechanical harvesting, and some possible differentially expressed proteins (DEPs) and metabolites (DEMs) related to carbohydrate metabolic pathways. Further identification of these differential molecules could enrich future research into the mechanisms of how different cultivation practices influence plant development and nutrient profiles, as well as their implications for insect growth regulation. This research provides valuable insights for refining mulberry variety breeding and optimizing cultivation varieties for the development of superior mulberry varieties for sericulture.

## Figures and Tables

**Table 1:** The annual leaf yield of two different cultivated varieties.

Varieties	10 May (Kg/Acre)	1 July (Kg/Acre)	30 August (Kg/Acre)	10 October (Kg/Acre)	Annual Leaf Yield (Kg/Acre)
ZJ1	923.93a	904.94b	960.82b	600.17b	3389.87b
N14	1295.01b	226.88a	320.64a	512.48a	2355.01a

Note: Data followed by different letters in each row are significantly different (*p* < 0.05, Tukey’s test).

**Table 2:** Comparison of nutrition components of two different cultivars.

Varieties	N14	ZJ1	*p*-Value
Water content (%)	73.61 ± 0.35	73.37 ± 1.18	0.24
Chlorophyll a content (mg.g^−1^)	8.08 ± 0.01	7.84 ± 0.01	0.02
Chlorophyll b content (mg.g^−1^)	2.81 ± 0.01	2.65 ± 0.01	0.05
Total protein content (mg.g^−1^)	22.76 ± 3.67	24.18 ± 2.86	0.34
Dietary fiber content (mg.g^−1^)	420.07 ± 6.12	413.65 ± 5.57	0.29
Ash content (mg.g^−1^)	129.30 ± 0.24	124.03 ± 0.85	0.05
Amino acid content (mg.g^−1^)			
Aspartic acid	19.32 ± 0.02	21.46 ± 0.09	0.06
Threonine	9.14 ± 0.07	9.78 ± 0.05	0.06
Serine	8.60 ± 0.08	9.63 ± 0.01	0.04
Glutamic acid	22.26 ± 0.12	26.04 ± 0.11	0.06
Proline	9.56 ± 0.07	10.67 ± 0.14	0.14
Glycine	11.43 ± 0.05	13.11 ± 0.05	0.09
Alanine	13.16 ± 0.02	14.43 ± 0.06	0.10
Valine	12.72 ± 0.13	13.90 ± 0.03	0.22
Methionine	1.45 ± 0.01	1.72 ± 0.01	0.13
Isoleucine	9.70 ± 0.11	11.84 ± 0.12	0.04
Leucine	18.53 ± 0.04	21.06 ± 0.06	0.10
Tyrosine	4.91 ± 0.01	6.33 ± 0.07	0.09
Phenylalanine	11.54 ± 0.03	13.34 ± 0.05	0.10
Histidine	4.63± 0.03	5.52 ± 0.04	0.25
Lysine	9.54 ± 0.12	10.56 ± 0.07	0.32
Arginine	9.40 ± 0.03	11.97 ± 0.22	0.22

**Table 3:** Larvae growth and cocoon yield of two groups on different cultivated leaves.

Groups	Stage	Larvae Number	Final Cocoon Number	Weight of 50 Whole Cocoons (g)	Weight of 50 Cocoon Shells (g)	Average Cocoon Shell Ratio (%)	Cocoon Shell Weight of Per 10,000 Larvae (g)
ZJ1	Spring	400	378.67 ± 8.74a	109.13 ± 0.92b	22.72 ± 0.36b	20.72 ± 0.19b	4261.45 ± 43.65b
	Autumn	400	371.33 ± 11.59a	94.67 ± 0.06a	19.43 ± 0.34a	20.53 ± 0.01a	3672.39 ± 77.89a
N14	Spring	400	381.33 ± 9.61a	110.42 ± 0.43c	22.54 ± 0.09b	20.41 ± 0.07a	4295.70 ± 10.34b
	Autumn	400	374.33 ± 4.73a	93.08 ± 0.91a	18.86 ± 0.38a	20.26 ± 0.02a	3555.25 ± 85.31a

Note: Data followed by different letters in each row are significantly different (*p* < 0.05, Tukey’s test).

## Data Availability

The raw data supporting the conclusions of this article will be available on request, without undue reservation.

## Supplementary Materials

The following supporting information can be downloaded at: https://www.mdpi.com/article/10.3390/ijms252413483/s1.

## Abbreviations

A/N-INV	alkaline/neutral invertase
DEP	differentially expressed protein
DEM	differentially expressed metabolite
FDR	false discovery rate
GO	gene ontology
iTRAQ	isobaric tags for relative and absolute quantitation
KEGG	Kyoto Encyclopedia of Genes and Genomes
rpS14	40 S ribosomal protein S14
sPLS-DA	squares-discriminant analysis model
SPS	sucrose phosphate synthase
SUS	sucrose synthesis
UDPG	uridine diphosphate glucose, UDP-glucose
